# SOCS2 correlates with malignancy and exerts growth-promoting effects in prostate cancer

**DOI:** 10.1530/ERC-13-0446

**Published:** 2014-04

**Authors:** Julia Hoefer, Johann Kern, Philipp Ofer, Iris E Eder, Georg Schäfer, Dimo Dietrich, Glen Kristiansen, Stephan Geley, Johannes Rainer, Eberhard Gunsilius, Helmut Klocker, Zoran Culig, Martin Puhr

**Affiliations:** 1 Experimental Urology, Department of UrologyInnsbruck Medical UniversityAnichstrasse 35A-6020, InnsbruckAustria; 1 Oncotyrol Laboratory for Tumor Biology and AngiogenesisInnsbruckAustria; 2 Institute of PathologyUniversity Hospital BonnBonnGermany; 3 Division of Molecular PathophysiologyInnsbruck Biocenter Medical University InnsbruckInnsbruckAustria

**Keywords:** prostate cancer, SOCS2, androgen, proliferation, survival

## Abstract

Deregulation of cytokine and growth factor signaling due to an altered expression of endogenous regulators is well recognized in prostate cancer (PCa) and other cancers. Suppressor of cytokine signaling 2 (SOCS2) is a key regulator of the GH, IGF, and prolactin signaling pathways that have been implicated in carcinogenesis. In this study, we evaluated the expression patterns and functional significance of SOCS2 in PCa. Protein expression analysis employing tissue microarrays from two independent patient cohorts revealed a significantly enhanced expression in tumor tissue compared with benign tissue as well as association with Gleason score and disease progression. *In vitro* and *in vivo* assays uncovered the involvement of SOCS2 in the regulation of cell growth and apoptosis. Functionally, *SOCS2* knockdown inhibited PCa cell proliferation and xenograft growth in a CAM assay. Decreased cell growth after SOCS2 downregulation was associated with cell-cycle arrest and apoptosis. In addition, we proved that SOCS2 expression is significantly elevated upon androgenic stimulation in androgen receptor (AR)-positive cell lines, providing a possible mechanistic explanation for high SOCS2 levels in PCa tissue. Consequently, SOCS2 expression correlated with AR expression in the malignant tissue of patients. On the whole, our study linked increased SOCS2 expression in PCa with a pro-proliferative role *in vitro* and *in vivo*.

## Introduction

Prostate cancer (PCa) represents the most common malignancy in men in the Western world (Jemal *et al*. 2011). The development and progression of PCa are strongly associated with the dysfunction of various signaling pathways. Preserved androgen signaling is one of the main characteristics of the disease, especially in advanced and castration-resistant PCa (Locke *et al*. 2008, Lonergan & Tindall 2011). Furthermore, cytokine and growth factor signaling cascades are frequently deregulated due to an altered expression of endogenous key regulators such as protein inhibitors of activated STAT (PIAS), Src-homology 2 (SH2)-containing protein tyrosine phosphatases, and suppressors of cytokine signaling (SOCS; Larsen & Ropke 2002, Heinrich *et al*. 2003, Tan & Nevalainen 2008, Dutt & Gao 2009, Puhr *et al*. 2009, Hoefer *et al*. 2012, Singh *et al*. 2012).

SOCS proteins have originally been described as the feedback inhibitors of cytokine-induced Janus kinase (JAK/STAT) signaling (Endo *et al*. 1997, Naka *et al*. 1997, Starr *et al*. 1997). However, they have also been reported to be associated with other pathways such as nuclear factor kappa B signaling (Ryo *et al*. 2003) and insulin signaling (Rui *et al*. 2002). The SOCS family consists of eight members: SOCS1–7 and cytokine-inducible SH2-containing protein (CIS). They contain an N-terminal region of variable length, a central SH2 domain, and a conserved C-terminal region called SOCS box. SOCS members can act as E3 ubiquitin ligases toward associated proteins (Kamura *et al*. 1998, 2004) and are rapidly induced upon the release of multiple growth factors and cytokines, with STAT molecules being the main transducers of these signals (Croker *et al*. 2008).

The involvement of SOCS1 and SOCS3 in PCa has been extensively investigated and both tumor suppressive and oncogenic roles have been reported (Bellezza *et al*. 2006, Neuwirt *et al*. 2007, 2009, Puhr *et al*. 2009, 2010, Pierconti *et al*. 2011). However, to date, only a limited number of studies have investigated the expression and functional significance of SOCS2 in PCa. In other malignancies, the upregulation or downregulation of SOCS2 has already been demonstrated (Arany *et al*. 2001, Schultheis *et al*. 2002, Wikman *et al*. 2002, Sutherland *et al*. 2004). Furthermore, *Socs2* knockout as well as transgenic mice have been reported to display gigantism (Metcalf *et al*. 2000, Greenhalgh *et al*. 2002), suggesting a dual role for SOCS2 in growth regulation. SOCS2 is preferentially upregulated by growth hormone (GH; Greenhalgh *et al*. 2002), erythropoietin (Starr *et al*. 1997, Wang *et al*. 2004), interleukins (ILs; Starr *et al*. 1997, Rico-Bautista *et al*. 2006), and prolactin (Pezet *et al*. 1999). Furthermore, SOCS2 is implicated in insulin-like growth factor (IGF) 1 and insulin signaling cascades (Dey *et al*. 1998, Sadowski *et al*. 2001). Deregulation of either of these signaling cascades has already been reported to be associated with PCa (Roberts 2004, Monti *et al*. 2007, Cox *et al*. 2009, Culig 2011, Goffin *et al*. 2011, Culig & Puhr 2012, Nakonechnaya *et al*. 2013). Therefore, we hypothesize that SOCS2 is a crucial regulator in PCa. To address this question, in this study, we investigated SOCS2 expression in tissue samples and carried out *in vitro* and *in vivo* assays to uncover its functional relevance in PCa. Our data clearly demonstrate a growth-promoting role for SOCS2 and provide an explanation for a high SOCS2 expression in malignant tissue via androgenic regulation.

## Materials and methods

### Tissue microarray and immunohistochemistry

A tissue microarray (TMA) (Innsbruck-TMA) containing tissue cores obtained from 90 PCa patients who underwent radical prostatectomy at the University Hospital Innsbruck was constructed and immunohistochemically stained as described elsewhere (Hoefer *et al*. 2012). In addition, SOCS2 expression in metastases was assessed using two TMAs constructed from tissue cores obtained from ten patients with lymph node and bone metastases respectively. Evaluation was done by an uropathologist (G S) using the semi-quantitative scoring system ‘quickscore’ (Detre *et al*. 1995). A second TMA (Bonn-TMA) comprising tissue specimens collected from 25 PCa patients who underwent radical prostatectomy at the University Hospital Bonn, Germany, was constructed and stained as described above. The study was approved by the institutional review boards. Staining intensity was evaluated by an independent pathologist (V S), defining a score between 0 (negative staining) and 3 (intense staining). The following antibodies were used: anti-SOCS2 (H-74) (1:100; Santa Cruz Biotechnology) and anti-androgen receptor (AR) (1:400; Epitomics, Burlingame, CA, USA).

### Methylation analysis

Bisulfite-converted DNA from FFPE tissue samples was prepared as described previously (Dietrich *et al*. 2012) (see Supplementary Materials and methods). Relative DNA methylation levels of the *SOCS2* gene locus referred to as total DNA as determined with a methylation-unspecific β-actin (*ACTB*) assay were assessed using a quantitative duplex PCR. PCR composition and thermal cycling were used as described previously (Dietrich *et al*. 2012).

### Cell culture and reagents

PC3, DU145, LNCaP, BPH-1, 22RV1, DUCaP, LAPC4, RWPE-1, and VCaP cells were obtained from the American Type Culture Collection (ATCC, Rockville, MD, USA) and cultured as indicated. The DUCaP-R subline was established by cultivation in the presence of 1 nM R1881 (Pfeiffer *et al*. 2010). The identity of the cell lines was confirmed by short tandem repeat analysis. Benign stromal PM151T and epithelial EP156T cells were cultured as described elsewhere (Kogan *et al*. 2006). For androgen stimulation, the cells were starved in RPMI containing 10% (v/v) charcoal-stripped FCS for 24 h and then treated with R1881 (DuPont NEN Products, Boston, MA, USA). To antagonize androgen-induced effects, LNCaP cells were incubated with 5 μM of bicalutamide for 24 h. LAPC4 cells, which are usually maintained in a medium containing dihydrotestosterone (DHT), were grown in RPMI containing 10% (v/v) charcoal-stripped FCS with or without 100 nM DHT (Sigma–Aldrich).

### Western blotting

Nuclear and cytoplasmic fractions were separated using the NE-PER Nuclear and Cytoplasmic extraction kit (Thermo Scientific, Rockford, IL, USA) following the manufacturer's instructions. Western blotting was carried out as described previously (Puhr *et al*. 2009). The following antibodies were used: anti-SOCS2 (1:500; Cell Signaling, Danvers, MA, USA), anti-GAPDH (1:100 000; Chemicon, Vienna, Austria), anti-cPARP (1:1000, Promega), and anti-Lamin A (1:5000, Abcam, Cambridge, UK).

### RNA isolation and qRT-PCR

RNA isolation, RT, and qRT-PCR were carried out as described elsewhere (Puhr *et al*. 2009) (see Supplementary Materials and methods).

### SOCS2 downregulation

PC3, DU145, and LNCaP cells were stably transfected with doxycycline-inducible shRNA constructs containing GFP against SOCS2 (shSOCS2-1 and shSOCS2-3) or luciferase (shLuc) (see Supplementary Materials and methods). The generation and transfection of constructs have been described in Sigl *et al*. (2009). For functional assays, the cells were pretreated with doxycycline (1 μg/ml) for 6 days to achieve SOCS2 downregulation.

### SOCS2 overexpression

pCMV6-AC-GFP and pCMV-AC-GFP-SOCS2 plasmids were obtained from Origene (Rockville, MD, USA). The cells were transfected with DNA (0.5 μg/six wells) using 3 μl of Fugene HD (Roche) for 48 h. For stable overexpression, the cells were selected with 0.5 mg/ml of G418 (neomycin) for 7 days.

### Proliferation measurement

Proliferation was assessed using [^3^H]thymidine incorporation assay as described in Hoefer *et al*. (2012). The medium was supplemented with 10% FCS. Viability was measured by WST assay. The cells were seeded in 96-well plates and grown in the medium. To the plates, 10 μl of WST reagent (Roche) were added for 60 min. Absorbance was assessed using Chameleon 5025 (HVD Life Sciences, Vienna, Austria).

### Clonogenic assay

A total of 1.500 cells (PC3 and DU145) or 10.000 cells (LNCaP) were seeded into a 75 cm^2^ cell-culture flask and incubated for 12 (PC3 and DU145) or 18 days (LNCaP). Colonies were stained as described in Hoefer *et al*. (2012).

### Apoptosis measurement and cell-cycle distribution

Apoptotic cells and cell-cycle distribution were assessed as described previously (Hoefer *et al*. 2012) and analyzed using the ModFit LT 3.2 software.

### Chick chorioallantoic membrane assay

Chick chorioallantoic membrane (CAM) assay was carried out as described (Deryugina & Quigley 2008) with slight modifications. For onplant preparation, native, nonpepsinized type I rat tail collagen (BD Bioscience, Schwechat, Austria) was neutralized with 0.2 M NaOH solution and mixed with 10× DMEM (Life Technologies Gibco). A total of 5×10^5^ pretreated PC3 cells were added to 50 μl of this solution. Collagen onplants with or without doxycycline (1 μg/ml) were applied to CAMs and incubated for 5 days. Xenografts were analyzed under a stereomicroscope with a digital camera (Olympus SZX10, Olympus E410, Vienna). The tumor area was determined using ImageJ (NIH). For histological analysis, onplants were excised from the CAMs, fixed in 4% paraformaldehyde, and processed for paraffin sectioning and immunohistochemistry (IHC). The following antibodies were used: anti-SOCS2 (H-74) (1:100; Santa Cruz) and anti-Ki67 (1:100; Dako, Glostrup, Denmark).

### Microarray dataset generation and analysis

The microarray dataset was generated at the Expression Profiling Unit of the Medical University Innsbruck following the manufacturer's protocols as described in Martowicz *et al*. (2013). The analysis of the in total 30 Affymetrix HuGene-1.0-ST-v1 GeneChips was carried out as described in Rainer *et al*. (2012) using functions from Bioconductor packages (Gentleman *et al*. 2004). The raw and pre-processed data were been submitted to GEO (accession number: GSE 50936).

### Statistical analyses

SPSS (V15.0) and GraphPad Prism 4 were used for statistical analyses. The Mann–Whitney *U* test or *t*-test was carried out to calculate significances between two groups. Correlation analysis was carried out using Pearson's method. Differences in relapse-free survival, defined as biochemical recurrence, were assessed using the Kaplan–Meier plots and log-rank test. Differences were considered statistically significant when *P* was <0.05.

## Results

### SOCS2 expression increases with malignancy and inversely correlates with time to disease recurrence

**Figure 1:**
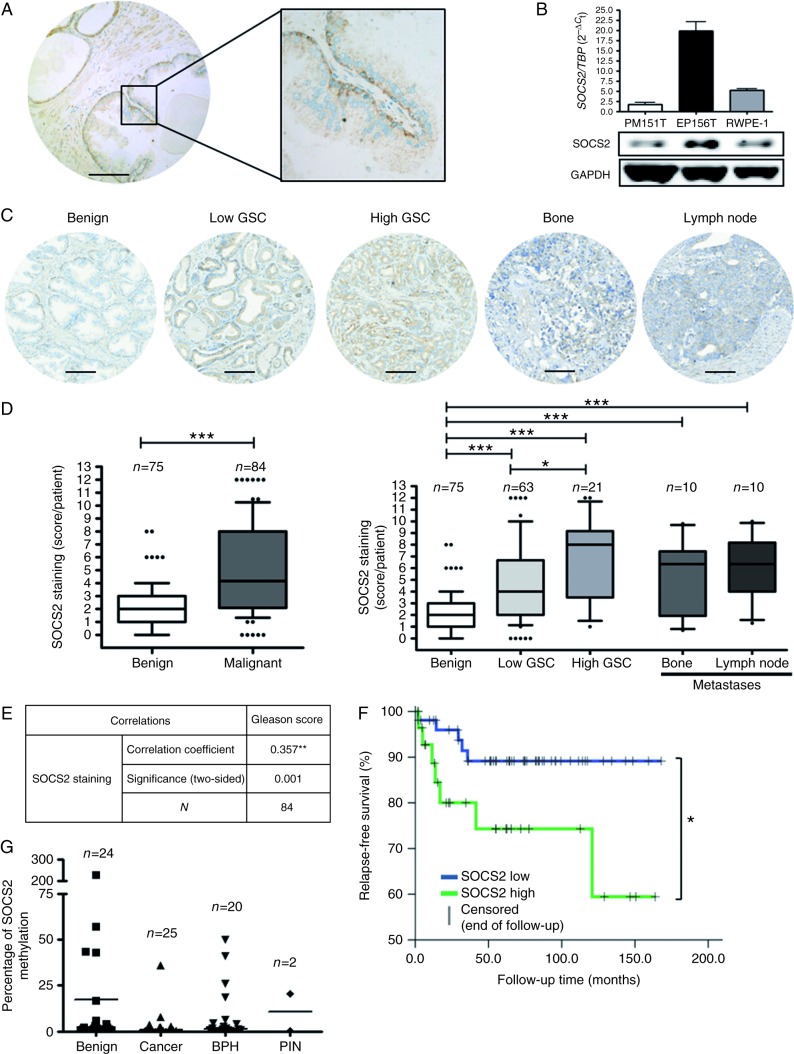
SOCS2 expression increases with malignancy and inversely correlates with relapse-free survival. (A) SOCS2 immunostaining of a benign prostate gland showing high SOCS2 expression in epithelial cells. Scale bar: 100 μm. (B) qRT-PCR data and representative western blots of three benign prostate cell lines: PM151T (stromal), EP156T (basal epithelial), and RWPE-1 (luminal epithelial). (C) Immunohistochemical SOCS2 staining of representative tissue samples of the Innsbruck-TMAs. Scale bar: 100 μm. (D) Statistical analysis of the Innsbruck-TMAs. Low Gleason: Gleason patterns 3+4 or below. High Gleason: Gleason patterns 4+3 or above. Box–Whiskers plot represents median values and 10–90 percentiles (*P<0.05 and ***P<0.001; Mann–Whitney *U* test). GSC, Gleason score. (E) Pearson's correlation analysis results for SOCS2 and Gleason score in malignant tissue samples of the TMA. (F) Kaplan–Meier curve assessment of relapse-free survival, defined as time to PSA progression, in patients exhibiting a low SOCS2 expression (*N*_(total)_=52; *N*_(relapse)_=5) vs patients exhibiting a high SOCS2 expression (*N*_(total)_=30; *N*_(relapse)_=7). High SOCS2 expression represents samples with SOCS2 staining score ≥6 according to the ‘quickscore method’ (*P<0.05; log-rank test). (G) Methylation status of the *SOCS2* gene locus was determined by qRT-PCR using methylation-specific primers. Results were normalized to β-actin and represent mean values.

To evaluate SOCS2 expression patterns in benign and malignant prostate tissue, we used a radical prostatectomy specimen TMA of two independent PCa cohorts. Ninety malignant and 79 benign cores of PCa specimens obtained in Innsbruck (Innsbruck-TMA) were evaluable after immunohistochemical staining. A representative benign gland (Fig. 1A) revealed a low SOCS2 expression in the surrounding stromal compartment (especially in smooth muscle fibers), whereas epithelial cells expressed higher levels of SOCS2. Within the epithelial compartment, the basal cell layer exhibited a much more intense SOCS2 staining than luminal cells. A similar phenomenon was observed in benign epithelial and stromal prostate cell lines. We detected the highest SOCS2 expression in EP156T cells, which predominantly represent a basal epithelial phenotype (Kogan *et al*. 2006), lower SOCS2 expression in the phenotypically luminal epithelial RWPE-1 cells, and the weakest SOCS2 expression in the stromal cell line PM151T (Fig. 1B). Quantitative analysis of the TMA demonstrated a significantly elevated SOCS2 expression in malignant areas compared with benign tissue, as well as in high- vs low-Gleason score samples (Fig. 1C and D). Furthermore, SOCS2 expression was significantly higher in bone and lymph node metastases compared with benign tissue (Fig. 1D). In primary tumor tissue, SOCS2 expression was positively correlated with the Gleason score (Fig. 1E), and relapse-free survival was significantly shorter in patients with a high SOCS2 expression (Fig. 1F). IHC staining of a second cohort of patients (Bonn-TMA) confirmed an elevated SOCS2 expression in low- as well as high-Gleason score tumors (Supplementary Figure 1, see section on supplementary data given at the end of this article). Testing for *SOCS2* gene hypermethylation, which is frequently reported for other tumor entities (Sutherland *et al*. 2004, Culig 2013), revealed no alteration in malignant tissue (Fig. 1G), consistent with the high expression of the protein in PCa.

### SOCS2 expression regulates PCa cell growth *in vitro* and *in vivo*

**Figure 2:**
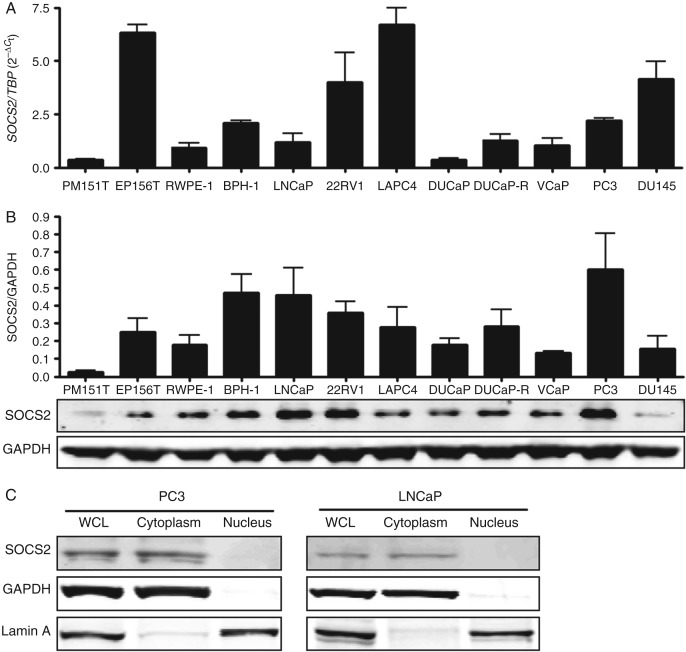
SOCS2 is expressed in the cytoplasm of prostate cell lines. (A) *SOCS2* mRNA and (B) SOCS2 protein expression in benign and malignant prostate cell lines was assessed by qRT-PCR and western blotting respectively. Data represent mean values of three independent experiments. (C) SOCS2 subcellular localization was determined by western blotting after cytoplasmic and nuclear fractionation of PC3 and LNCaP cell lysates.

**Figure 3:**
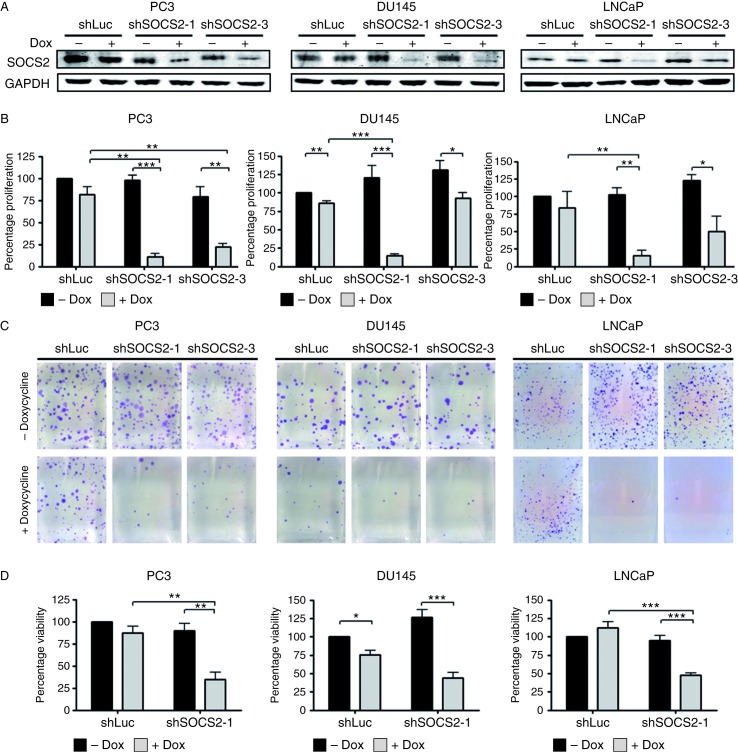
SOCS2 downregulation decelerates cell growth. (A) SOCS2 downregulation after stable transfection of PC3, DU145, and LNCaP cells with doxycycline-inducible shRNA sequences against SOCS2 (shSOCS2-1 and shSOCS2-3) or luciferase (shLuc). A representative western blot after 6 days of doxycycline treatment is shown. (B) Proliferation after SOCS2 downregulation was assessed by the measurement of [^3^H]thymidine incorporation. Data represent means±s e m. from three independent experiments (*P<0.05; **P<0.01; and ***P<0.001; *t*-test). (C) Colony formation ability was measured by clonogenic assay. (D) Viability after SOCS2 downregulation was measured using WST reagent. Data represent means±s e m from three independent experiments (*P<0.05; **P<0.01; and ***P<0.001; *t*-test).

All the tested prostate cell lines expressed both SOCS2 mRNA and protein, as measured by qRT-PCR and western blotting respectively (Fig. 2A and B). The observed discrepancy between mRNA and protein levels indicates a tight regulation of SOCS2 protein stability via proteasomal degradation. Cellular fractionation and subsequent western blotting revealed SOCS2 expression predominantly in the cytoplasmic fraction (Fig. 2C). To uncover a possible influence of SOCS2 on cell growth, we carried out functional assays following SOCS2 depletion. For this purpose, PC3, DU145, and LNCaP cells were stably transfected with doxycycline-inducible SOCS2 shRNA constructs and treated with doxycycline for 6 days to achieve prolonged SOCS2 downregulation (Fig. 3A). Subsequent [^3^H]thymidine incorporation and clonogenic assays revealed a significant decrease in both proliferation (Fig. 3B) and colony formation ability (Fig. 3C) in cells in which *SOCS2* was silenced. We obtained similar results with both specific shRNA sequences; however, growth inhibition with shSOCS2-1 was more prominent. The WST assay confirmed decreased cell viability after SOCS2 downregulation (Fig. 3D). In line with these findings, SOCS2 overexpression increased the proliferation and clonogenic potential in PC3 cells (Supplementary Figure 2, see section on supplementary data given at the end of this article).

**Figure 4:**
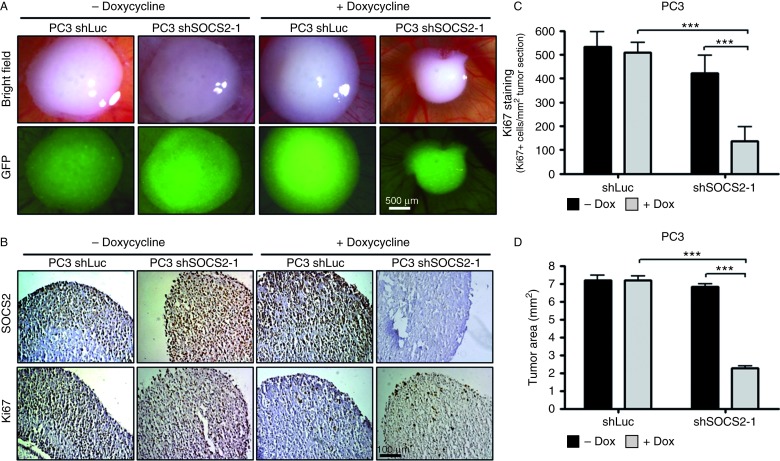
SOCS2 expression influences tumor growth *in vivo*. (A) Bright-field and fluorescence pictures of representative tumors obtained in a CAM assay. Before engraftment, the cells were pretreated with or without doxycycline for 6 days. (B) Immunohistochemical staining of tumor cross-sections for Ki67 and SOCS2. (C and D) Statistical analysis of Ki67-positive cells/cm^2^ tumor cross-section (C) and tumor area (D) of the CAM onplants. Data represent means±s.d. of five onplants/treatment, done in two independent experiments (*P<0.05 and ***P<0.001; *t*-test).

To confirm a possible anti-proliferative effect of *SOCS2* knockdown on tumor growth *in vivo*, we applied a CAM assay using PC3 shSOCS2-1 or shLuc cells. In the past three decades, the CAM assay has become an accepted and reliable *in vivo* model to replace animal experiments for testing different treatments (Armstrong *et al*. 1982, Kunzi-Rapp *et al*. 2001, Taizi *et al*. 2006, Tavaszi & Budai 2006, Saw *et al*. 2008). In xenografts derived from *SOCS2* knockdown cells, we observed a significantly reduced tumor area after 5 days on the CAM (Fig. 4A and D). Immunohistochemical staining revealed a reduced SOCS2 expression in the tumor cells as well as a significant decrease in the proportion of Ki67-positive cells (Fig. 4B and C).

### *SOCS2* knockdown leads to cell-cycle arrest and increased apoptosis

**Figure 5:**
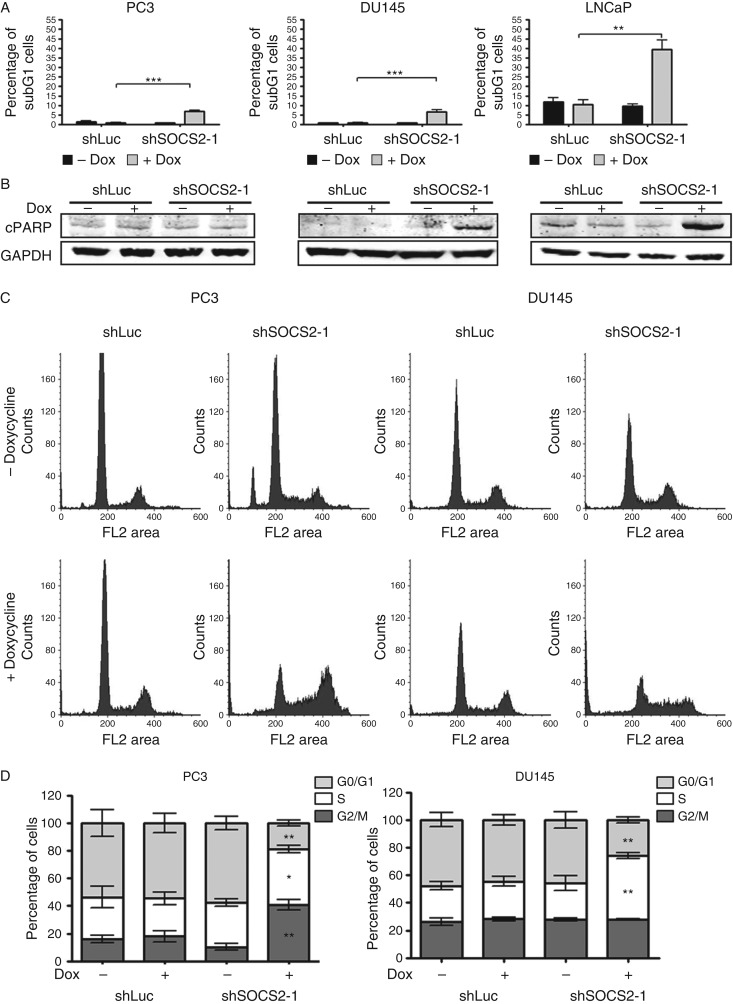
SOCS2 expression influences apoptosis and cell-cycle progression. (A and B) Apoptosis after SOCS2 downregulation was assessed by flow cytometry after PI staining (A), as well as cPARP measurement by western blotting (B) after 6 days of doxycycline treatment. Data represent means±s.e.m. from three independent experiments (**P<0.01 and ***P<0.001; *t*-test). (C) Representative histograms and (D) statistical analysis of cell-cycle distribution after SOCS2 downregulation as measured by flow cytometry. Data represent means±s.e.m. from three independent experiments (**P<0.01; and ***P<0.001; *t*-test).

To elucidate the mechanism underlying the decreased cell growth after SOCS2 downregulation, we measured apoptosis and cell-cycle distribution. The percentage of apoptotic cells was slightly increased in PC3 and DU145 cells after *SOCS2* silencing compared with the control cells. However, LNCaP cells were more sensitive and displayed a 40% apoptosis rate after *SOCS2* knockdown (Fig. 5A). These findings were confirmed by the measurement of cleaved PARP (cPARP) levels by western blotting. As expected, LNCaP shSOCS2 cells exhibited a massive increase in cPARP levels compared with the respective controls, while this effect was less distinct in PC3 and DU145 cells (Fig. 5B). To further uncover the molecular basis for altered cell proliferation after *SOCS2* knockdown, we analyzed cell-cycle distribution in the control as well as shSOCS2 cells. As has been mentioned above, LNCaP cell line responded with a massive increase in the subG1 proportion, which represents the apoptotic population (Supplementary Figure 3, see section on supplementary data given at the end of this article). However, we observed an altered cell-cycle distribution pattern in PC3 and DU145 cells after SOCS2 downregulation compared with the control cells (Fig. 5C). Statistical analysis revealed an S-phase growth arrest in SOCS2-depleted PC3 and DU145 cells and in addition an increase in the percentage of G2/M-phase cells in PC3 shSOCS2 cell line. The G0/G1-phase proportion was significantly decreased in both cell lines after *SOCS2* knockdown (Fig. 5D).

### SOCS2 expression is upregulated by androgens

**Figure 6:**
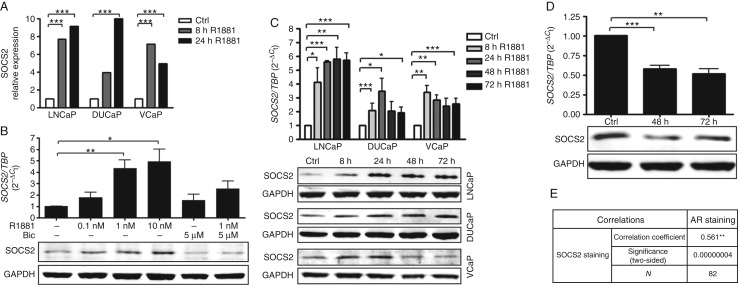
SOCS2 is upregulated upon androgenic stimulation. (A) Affymetrix data of R1881-treated LNCaP, DUCaP, and VCaP cells (***P<0.001; *t*-test). (B) qRT-PCR data and representative western blot of LNCaP cells treated with increasing concentrations of R1881 and bicalutamide (Bic) or a combination of both for 24 h. Data represent mean values of three independent experiments (*P<0.05; **P<0.01; *t*-test). (C) qRT-PCR data and representative western blots of LNCaP, DUCaP, and VCaP cells treated with 1 nM of R1881 for 8, 24, 48, and 72 h. Data represent mean values of three independent experiments (*P<0.05; **P<0.01; and ***P<0.001; *t*-test). (D) qRT-PCR data and representative western blot of LAPC4 cells grown in steroid-free medium with the addition of 100 nM dihydrotestosterone (DHT) (ctrl) or after 24 or 48 h of DHT depletion. Data represent mean values of three independent experiments (**P<0.01; ***P<0.001, *t*-test). (E) Pearson's correlation analysis results for SOCS2 and AR staining in malignant tissue samples of the TMA.

Finally, we aimed to provide a possible explanation for the significantly increased SOCS2 expression in the malignant tissue of PCa patients. AR is able to activate STAT5 (Tan *et al*. 2008), which in turn has been shown to upregulate SOCS2 in head-and-neck squamous cell carcinoma (HNSCC; Sen *et al*. 2011). Therefore, we hypothesized that SOCS2 expression might be influenced upon androgenic stimulation in PCa. To address this issue, we used Affymetrix GeneChip expression data of LNCaP, DUCaP, and VCaP cells cultured in the absence or presence of R1881. *SOCS2* was identified as an androgen-regulated gene (Fig. 6A). We confirmed this finding also at the mRNA and protein levels, demonstrating androgenic SOCS2 upregulation in a dose-dependent manner in LNCaP cells. Furthermore, treatment with the anti-androgen bicalutamide was sufficient to reverse this effect (Fig. 6B). Androgen-induced SOCS2 expression increase was in addition time dependent and peaked between 8 and 24 h in LNCaP, DUCaP, and VCaP cells (Fig. 6C). To further confirm androgen responsiveness of SOCS2, we expected that androgen withdrawal would result in a diminished SOCS2 expression. Hence, we depleted LAPC4 cells, which are usually grown in a medium containing DHT. As expected, we observed a significantly decreased SOCS2 expression in cells grown in the steroid-free medium, compared with cells cultured in the presence of DHT (Fig. 6D). Finally, we were able to translate these findings to the patients, demonstrating a significant correlation between SOCS2 and AR expression in patient samples (Fig. 6E).

## Discussion

Deregulation of endogenous feedback inhibitors of JAK/STAT signaling is well recognized in PCa and other cancers. We have previously demonstrated that SOCS1, SOCS3, and PIAS1 are upregulated in prostate tumors compared with benign tissue and have fundamental roles in proliferation and apoptosis (Neuwirt *et al*. 2009, Puhr *et al*. 2009, Hoefer *et al*. 2012). SOCS2 is hypermethylated in ovarian and breast cancers, in which its reduced expression is associated with the activation of STAT3, indicating an increased cytokine responsiveness in these tumors (Sutherland *et al*. 2004). *SOCS2* mRNA has been found to be downregulated in pulmonary cancer, hepatocellular cancer and PCa (Wikman *et al*. 2002, Hendriksen *et al*. 2006, Qiu *et al*. 2013). On the other hand, *SOCS2* acts as an oncogene in advanced stages of chronic myeloid leukemia as well as in precursors of anal cancer, where it is significantly upregulated (Arany *et al*. 2001, Schultheis *et al*. 2002). However, other studies that have investigated SOCS2 in PCa have addressed different aspects of SOCS2 function and have been carried out under different experimental conditions (Hendriksen *et al*. 2006, Iglesias-Gato *et al*. 2013, Zhu *et al*. 2013).

In the present study, we investigated SOCS2 expression patterns in benign and low- and high-Gleason score tumor samples as well as in prostate cell lines. Differences between SOCS2 mRNA and protein levels observed in the cell lines are not surprising, because SOCS2 protein is highly unstable as a consequence of active degradation (Siewert *et al*. 1999). In tissue samples, we detected significantly increased SOCS2 levels in malignant areas and were furthermore able to positively correlate SOCS2 expression with increasing Gleason scores. In addition, we could demonstrate that patients with a high SOCS2 expression are more likely to experience biochemical tumor relapse. These findings are partially in line with those of a recent study (Zhu *et al*. 2013), which identified an increased SOCS2 expression in malignant vs benign tissue. However, in contrast to our findings, that study observed a significant upregulation only in low-Gleason score tumors, whereas in high-Gleason score tumors only a trend toward a high SOCS2 expression was observed. SOCS2 expression was found to be associated with longer relapse-free survival. Besides ethnic differences, a diverse designation of tumors into low- and high-Gleason score groups, as well as different IHC scoring methods, might explain these discrepancies. As *SOCS2* is an androgen-regulated gene, ethnic differences between the study populations may be important for the analysis of data. Although more studies comparing AR target gene expression in different ethnic groups have to be carried out, one can expect that differences occur in certain tumor subgroups as a consequence of dissimilar AR transcriptional activities. Nevertheless, the generally elevated SOCS levels found in PCa were consistently observed in two independent patient cohorts. Furthermore, it should be mentioned that Iglesias-Gato *et al*. have very recently reported a higher SOCS2 immunoreactivity in parallel with a Gleason score increase in a Swedish population (2013).

In concordance with the tissue expression profile, our functional assays point to a potential growth-promoting activity of SOCS2 in PCa. First, SOCS2 downregulation leads to a significantly diminished tumor growth *in vivo*. Secondly, *SOCS2* knockdown results in a substantial decrease in cell proliferation and clonogenicity in AR-negative and -positive cell lines. We could link this growth inhibition to S-phase and G2/M-phase cell-cycle arrests with a slight increase in apoptosis in AR-negative PC3 and DU145 cells. Interestingly, SOCS2-depleted LNCaP cells exhibit a dramatic increase in apoptosis, suggesting an additional role for SOCS2 in AR-positive cells, which renders them more sensitive to *SOCS2* knockdown.

The concept of SOCS2 as a growth promoter rather than as an inhibitor is supported by data obtained from other studies. Both *Socs2* knockout and transgenic mice display gigantism (Metcalf *et al*. 2000, Greenhalgh *et al*. 2002). This observation can be explained by the circumstance that SOCS2 plays a dual role in growth regulation, depending on its concentration. At low levels, SOCS2 inhibits several cascades such as GH, prolactin, and IL signaling, whereas at high levels, SOCS2 restores or potentiates responsiveness to these growth factors (Favre *et al*. 1999, Pezet *et al*. 1999, Tannahill *et al*. 2005, Piessevaux *et al*. 2006). In this context, SOCS2 has been demonstrated to enhance GH, IL2, and IL3 signals and subsequent proliferation via proteasomal degradation of other SOCS members, thereby overcoming their inhibitory effects (Favre *et al*. 1999, Tannahill *et al*. 2005). These findings suggest SOCS2 to be a positive regulator of proliferation when expressed at high levels. PCa is characterized by the deregulation of several signaling pathways including GH, prolactin, IL6, and the IGF/insulin axis (Roberts 2004, Monti *et al*. 2007, Cox *et al*. 2009, Culig 2011, Goffin *et al*. 2011, Culig & Puhr 2012, Nakonechnaya *et al*. 2013), which can induce SOCS2 expression (Trengove & Ward 2013). We therefore hypothesize that in PCa SOCS2 is generally highly expressed as a consequence of these oncogenic events and thus acts as an accelerator of proliferative signals in this disease. Hence, it is not surprising that *SOCS2* knockdown in PCa cells resulted in growth inhibition.

In addition, we proved that SOCS2 is upregulated by androgens in AR-positive cell lines. SOCS2 is induced upon androgenic stimulation in a dose- and time-dependent manner at both mRNA and protein levels. Androgen responsiveness of SOCS2 was further confirmed in an experimental setting, where androgens were depleted. Although we cannot exclude alternative mechanisms, a regulation via STAT5 might be a possible explanation. AR increases the transcriptional activity of STAT5 and *vice versa* (Tan *et al*. 2008). STAT5 has been shown to stimulate SOCS2 expression in HNSCC (Sen *et al*. 2011). Furthermore, SOCS2 has been identified as a direct STAT5 target in the liver (Vidal *et al*. 2007). On the basis of these data and our results, we hypothesize that in AR-positive cells SOCS2 expression is increased due to STAT5 activation following androgen stimulation. This mechanism provides a possible explanation for the elevated SOCS2 expression in prostate tumors, which frequently harbor a highly active androgen signaling (Buchanan *et al*. 2001, Lonergan & Tindall 2011).

While this manuscript was in preparation, Iglesias-Gato *et al*. (2013) reported that SOCS2 antagonizes the oncogenic events caused by GH in PCa. It should be mentioned that differences in the results of the two studies may be explained by the presence or absence of GH in the experiments carried out. In the present study, all the experiments were carried out in cells not treated with GH. On the whole, our study is the first to link increased SOCS2 expression in tissue samples of PCa patients with a growth-promoting role for SOCS2 *in vitro* and *in vivo*. Furthermore, we demonstrated androgen responsiveness of SOCS2 in AR-positive cell lines, providing a mechanistic explanation for the high SOCS2 expression in PCa via androgenic stimulation. These findings render SOCS2 an interesting candidate for further investigations to clarify specific pathways involved in SOCS2-mediated effects on PCa cell growth.

## Supplementary data

This is linked to the online version of the paper at http://dx.doi.org/10.1530/ERC-13-0446.

## Author contribution statement

J Hoefer carried out all the *in vitro* experiments except the Affymetrix analysis and wrote the manuscript. J Kern and E Gunsilius carried out and analyzed the CAM assay. P Ofer established shSOCS2 and shLuc LNCaP cell lines. I E Eder and J Rainer conducted the Affymetrix experiment. G Schäfer scored IHC of the Innsbruck-TMA in cooperation with MP. D Dietrich and G Kristiansen planned and coordinated the methylation analysis and IHC staining of the Bonn-TMA. S Geley cloned the plasmids for shRNA transfection. H Klocker was responsible for the generation of the Innsbruck-TMA. Z Culig supervised J Hoefer and helped writing the manuscript. M Puhr supervised J Hoefer, established shSOCS2 and shLuc PC3 and DU145 cell lines, organized the *in vivo* experiments, analyzed the Innsbruck-TMA and helped writing the manuscript. In addition, all the co-authors improved the manuscript and approved its final version. M Puhr and Z Culig are joint corresponding authors.

## Supplementary Material

Supplementary Data
