# Supplementary material for: SOCS2 correlates with malignancy and exerts growth-promoting effects in prostate cancer
Source: Endocr Relat Cancer. 2013 Nov 26;21(2):175–87. doi: 10.1530/ERC-13-0446 (PMC3907181; doi:10.1530/ERC-13-0446)
Supplement: Supplementary Data [file supp_21_2_175__index.html]

SOCS2 correlates with malignancy and exerts growth-promoting effects in prostate cancer — Oncogenic role of SOCS2 in PCa — Supplementary Data 

# SOCS2 correlates with malignancy and exerts growth-promoting effects in prostate cancer

## Supplementary Data

**Files in this Data Supplement:**

- Supplementary Figure 1 - Statistical analysis and representative pictures of the Bonn-TMA. Low Gleason: Gleason pattern 3+4 or below. High Gleason: Gleason patterns 4+3 or above (\*, p<0.05; \*\*\*, p< 0.001, Mann-Whitney-Test). Scalebar: 300 μm. (PDF 74 KB)
- Supplementary Figure 2 - **SOCS2 overexpression increases cell growth. (A)** For over-expression, PC3 cells were transfected with pCMV6-AC-GFP-SOCS2 or empty vector (EV) and selected with G418 for 8 days. Proliferation and colony formation were measured by [3H]thymidine incorporation and clonogenic assay, respectively. Data represent mean ± SEM from 3 independent experiments (\*, p<0.05; \*\*, p<0.01; \*\*\*, p< 0.001, t-Test). **(B)** SOCS2 overexpression control by Western blot after transient transfection for 48 h. (PDF 314 KB)
- Supplementary Figure 3 - Representative FACS plots of cell cycle distribution after SOCS2 down-regulation in LNCaP cells as measured by flow cytometry. (PDF 148 KB)
- Supplementary Information 1 - (PDF 9 KB)
