# Supplementary figures and images for: SOCS2 correlates with malignancy and exerts growth-promoting effects in prostate cancer
Source: Endocr Relat Cancer. 2013 Nov 26;21(2):175–87. doi: 10.1530/ERC-13-0446 (PMC3907181; doi:10.1530/ERC-13-0446)

# Hoefer et al. Figure S1

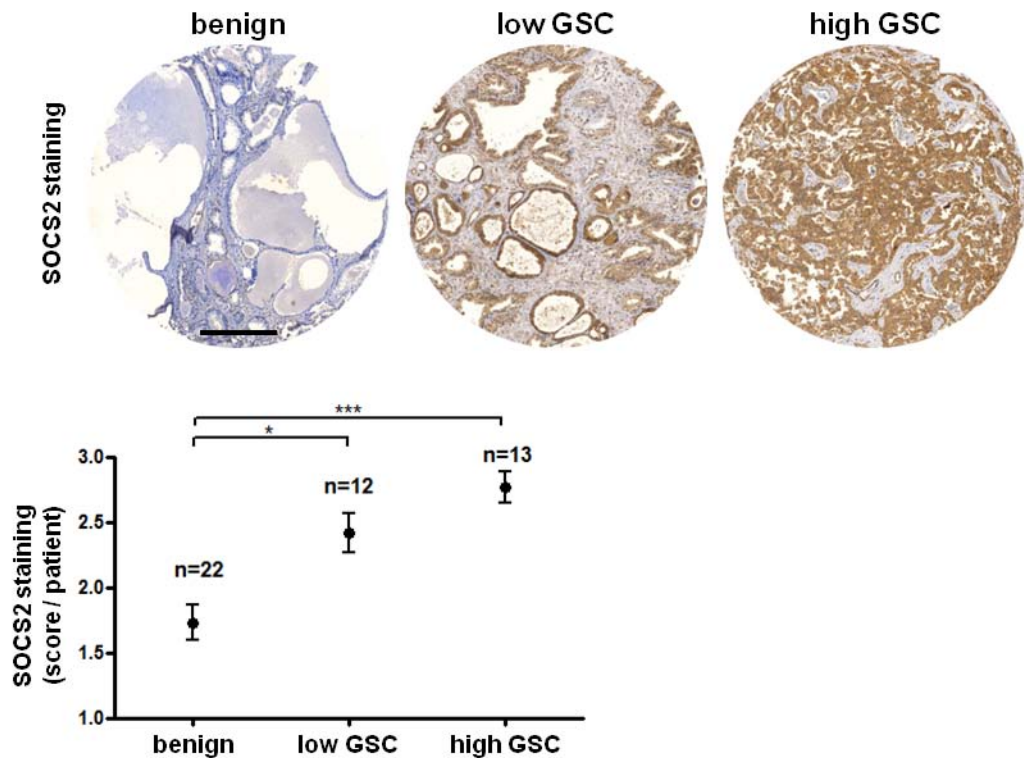

Supplement: Supplementary Data [file supp_ERC-13-0446_Supplementary_figure_1.pdf]

# Hoefer et al. Figure S2

**A**

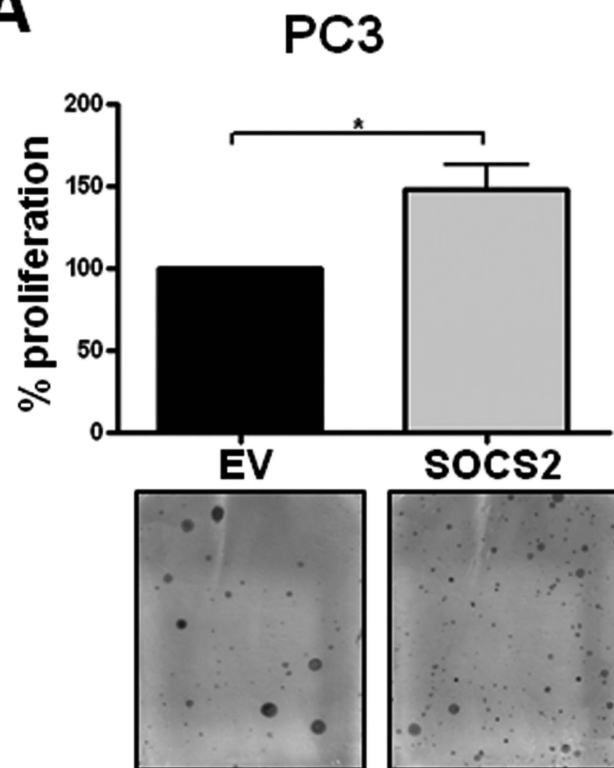

**B**

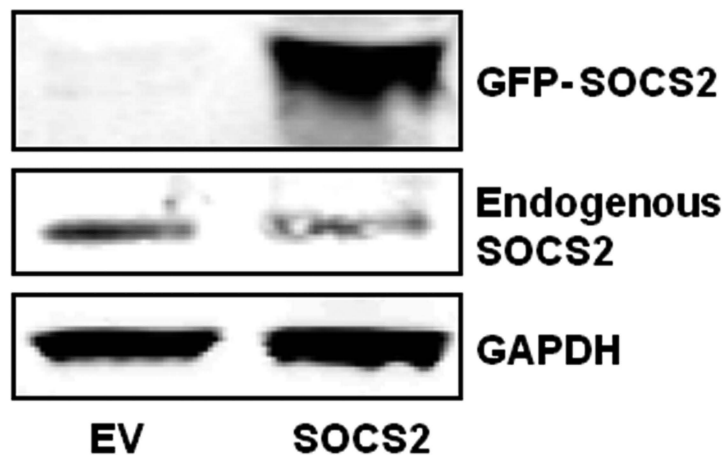

Supplement: Supplementary Data [file supp_ERC-13-0446_Supplementary_figure_2.pdf]

# Hoefer et al. Figure S3

LNCaP

shLuc

shSOCS2-1

- Doxycycline

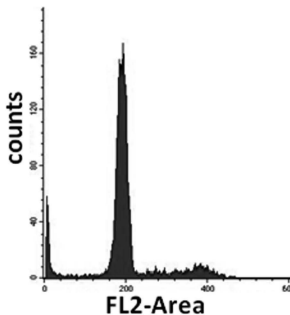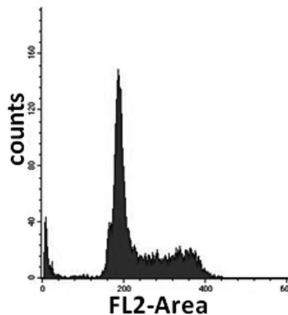

+ Doxycycline

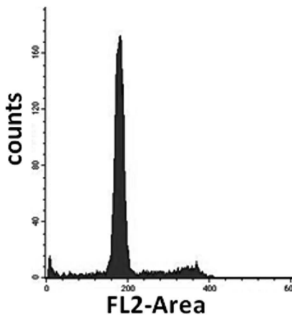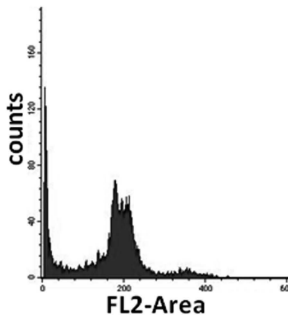

Supplement: Supplementary Data [file supp_ERC-13-0446_Supplementary_figure_3.pdf]
