# Supplementary material for: SOCS2 correlates with malignancy and exerts growth-promoting effects in prostate cancer
Source: Endocr Relat Cancer. 2013 Nov 26;21(2):175–87. doi: 10.1530/ERC-13-0446 (PMC3907181; doi:10.1530/ERC-13-0446)
Supplement: Supplementary Data [file supp_ERC-13-0446_Supplementary_information_1.pdf]

Sequences of the used shRNA, primers, probes

shRNA sequences (sense, antisense sequences targeting SOCS2 or luciferase are underlined)

shSOCS2-1

GATCCCCGAAGCCAAAGAGAAATTAATTCAAGAGATTAATTTCTCTTTGGCTTCTTTTTGGAAA

shSOCS2-3

GATCCCCGCTAAAGACAAGAATATCATTCAAGAGATGATATTCTTGTCTTTAGCTTTTTGGAAA

shLuc

GATCCCCCTTACGCTGAGTACTTCGATTCAAGAGATCGAAGTACTCAGCGTAAGTTTTTGGAAA

Primer and probes for qRT-PCR

SOCS2 forward

CAGATGTGCAAGGATAAGCGG

SOCS2 reverse

CAGATAAAGGTGAACAGTGCCG

SOCS2 probe

6-FAM-CAGGTCCAGAAGCCCCCCGG-TAMRA

TBP forward

5´-CACGAACCACGGCACTGATT-3´;

TBP reverse

TTTTCTTGCTGCCAGTCTGGAC

TBP probe

6-FAM-TCTTCACTCTTGGCTCCTGTGCACA-TAMRA

Primers and probes for methylation analysis

SOCS2 forward

AATTAAACTCCCGAACTCGAA

SOCS2 reverse

GTTGGGTTTGCGGGGTTC

SOCS2 probe

6-FAM-AAACGTCGAACGCCTCCC-TQ2

ACTB forward

CCAATAAAACCTACTCCTCCCTTAA

ACTB reverse

GTGATGGAGGAGGTTTAGTAAGTT

ACTB probe

R6G-ACCACCACCCAACACACAATAACAAACACA-BBQ-650
